# HIV-1 late diagnosis in Cabo Verde: associated factors and implications for preventive strategies

**DOI:** 10.3389/fepid.2026.1861884

**Published:** 2026-06-11

**Authors:** Silvânia da Veiga Leal, Mafalda N. S. Miranda, Ricardo Parreira, Victor Pimentel, Isabel Inês Monteiro de Pina Araújo, Marta Pingarilho, Ana B. Abecasis

**Affiliations:** 1 Instituto Nacional de Saúde Pública de Cabo Verde, Largo do Desastre da Assistência, Chã de Areia, Praia, Cabo Verde; 2 Global Health and Tropical Medicine (GHTM), Associate Laboratory in Translation and Innovation Towards Global Health (LA-REAL), Instituto de Higiene e Medicina Tropical (IHMT), Universidade NOVA de Lisboa, Lisbon, Portugal; 3 Faculdade de Ciências e Tecnologia, Universidade de Cabo Verde, Campus do Palmarejo Grande, Praia, Cabo Verde; 4 One Health Research Center-Cabo Verde (NEST-CV), Universidade de Cabo Verde, Campus do Palmarejo Grande, Praia, Cabo Verde

**Keywords:** associated factors, Cabo Verde, CD4+ T lymphocyte, epidemiology, genetic, HIV-1 infection, late HIV diagnosis

## Abstract

**Background:**

Late diagnosis of HIV infection remains a major public health challenge, associated with higher morbidity and mortality, ongoing transmission, opportunistic infections, and higher healthcare costs. Despite free access to HIV-1 testing and antiretroviral therapy in Cabo Verde, the prevalence and determinants of late HIV diagnosis remain poorly characterised. Therefore, this study aimed to assess the prevalence of late HIV diagnosis and identify associated factors among newly diagnosed, treatment-naïve individuals with HIV-1 in Cabo Verde.

**Methods:**

A cross-sectional study combining interview - based data with retrospective extraction of clinical data was conducted among 135 newly diagnosed, treatment - naïve individuals between 2018 and 2019 across four islands of Santiago, Fogo, São Vicente, and Boavista. Late HIV diagnosis was defined as CD4+ < 350 cells/µL at diagnosis or the presence of an AIDS-defining condition. Sociodemographic and clinical data were collected, and viral genetic diversity was assessed through nucleotide ambiguity analysis. Associations with late HIV diagnosis were evaluated using univariable and multivariable logistic regression, presented as odds ratios (OR) and 95% confidence intervals (CI).

**Results:**

Overall, 56.3% (76/135) of individuals were classified as having late HIV diagnosis. In multivariable analysis, being single (aOR: 2.79; 95% CI: 1.13–6.92; *p* = 0.027) and having viral load ≥5.0 log copies/mL (aOR: 5.24; 95% CI: 1.80–15.25; *p* = 0.002) were independently associated with late HIV diagnosis. Genetic ambiguity analysis showed a predominance of recent infections (68.3%), with low concordance with CD4+ T-cell counts.

**Conclusions:**

HIV-1 late diagnosis remains highly prevalent in Cabo Verde and is associated with single marital status and higher viral load. These findings highlight persistent gaps in early HIV testing and timely linkage to care. Strengthening targeted testing strategies is essential to improve clinical outcomes and reduce HIV transmission, in line with the UNAIDS 95-95-95 targets.

## 1 Introduction

The HIV/AIDS pandemic remains a significant challenge to global public health. The introduction of antiretroviral therapy (ART) has significantly increased life expectancy among people living with HIV (PLHIV) by reducing mortality and morbidity associated with HIV/AIDS. In 2015, the World Health Organization (WHO) recommended universal ART initiation for all PLHIV, regardless of their clinical or immune status (1, 2). Nevertheless, despite these recommendations, a substantial proportion of individuals continue to be diagnosed late, often presenting with advanced disease. This highlights ongoing challenges in early diagnosis and treatment access, which complicates treatment outcomes and increases the risk of onward transmission (2).

Late diagnosis of HIV infection remains a major barrier to achieving the global HIV targets established by UNAIDS. While the 90-90-90 targets were set for 2020, they were followed by the more ambitious 95-95-95 targets for 2030, which emphasise early diagnosis, timely initiation of antiretroviral therapy, and sustained viral suppression (3, 4). However, recent global estimates indicate that these targets have not yet been fully achieved. In 2024, an estimated 87% of PLHIV were aware of their HIV status, 89% were receiving ART, and 94% of those on treatment had achieved viral suppression (5).

Despite significant efforts to establish strategies to improve early diagnosis of HIV infection, the prevalence of individuals diagnosed late remains a significant problem worldwide affecting both high- and low-income settings (6–9). Studies indicate that the prevalence of late HIV diagnosis varies widely, ranging from 15% to 66% in Europe (10–12), 72% to 83% in Asia (13), and 35% to 90% in Africa (14–17). Late HIV diagnosis is associated with serious consequences, including increased mortality, higher risk of drug resistance, and greater healthcare costs (18–22).

Late diagnosis of HIV infection is increasingly recognised as a key public health indicator, reflecting delays in HIV testing and missed opportunities for earlier detection. According to the updated definition proposed by Croxford et al. (23) late HIV diagnosis is defined as a diagnosis with a CD4+ T-lymphocyte count below 350 cells/μL at the time of diagnosis or the presence of an AIDS-defining condition, regardless of CD4+ T-cell count. This concept emerged in response to the limitations of the late presentation definition proposed by the European Late Presenter Consensus Working Group, which is primarily based on immunological and clinical criteria at the time of diagnosis and may not adequately reflect the timing of infection or distinguish between delayed diagnosis and inter-individual variability in disease progression (24, 25). As such, the concept of late HIV diagnosis provides a more appropriate framework for assessing delays in HIV testing and gaps in access to care.

The CD4+ T lymphocyte count at diagnosis remains the most widely used biomarker to estimate the time elapsed between HIV infection and detection (26–28). However, CD4+ T lymphocytes counts vary significantly between individuals and over time, reflecting biological and immunological differences. In this context, viral genetic diversity emerges as a complementary marker. In most cases, infection is established by a single founder virus (29), which evolves rapidly in untreated individuals, accumulating mutations that progressively increase viral diversity (29–32). Viral diversity tends to increase linearly during the early stages of infection, later reaching a plateau and potentially decreasing in advanced stages of the disease (31, 33). Analysis of nucleotide ambiguity in HIV sequences thus allows indirect estimation of infection duration, providing information complementary to that obtained from CD4+ T lymphocytes counts (34–36).

Cabo Verde has seen significant progression in its national response to the HIV epidemic since the first cases were identified in 1986. The introduction of free ART in 2004 marked a key milestone, increasing treatment coverage and reducing mortality associated with HIV infection. Currently, HIV diagnosis and treatment are free and accessible nationwide, with ART initiation immediately after diagnosis, in accordance with WHO guidelines. Clinical monitoring includes biannual CD4+ T lymphocyte counts and annual viral load assessments (37, 38). Significant efforts have been implemented to strengthen early diagnosis and prompt initiation of ART, contributing to a reduction in HIV prevalence to 0.6%, while maintaining mother-to-child transmission rates below 2% (37, 39, 40).

Early diagnosis and timely access to HIV treatment are crucial to maximising individual health outcomes and reducing HIV-related morbidity and mortality. The only study conducted on the largest island of Santiago, between 2004 and 2011, revealed that 51.9% of individuals were diagnosed late, highlighting the urgent need for more effective strategies to promote early diagnosis (16).

To better understand late diagnosis among new HIV-1 diagnoses in Cabo Verde, we conducted a retrospective analysis of treatment-naïve individuals diagnosed between 2018 and 2019 across the islands of Santiago, Fogo, São Vicente, and Boavista. The combination of CD4+ T lymphocyte counts and nucleotide ambiguity allowed for a more precise characterization of late HIV diagnosis, providing valuable insights to support the development of early diagnosis and prevention strategies. These islands account for more than 85% of PLHIV in the country and represent priority settings for epidemiological surveillance and public health interventions (37).

## 2 Materials and methods

### 2.1 Study population

The study included treatment-naïve individuals recently diagnosed with HIV-1, between 2018 and 2019, recruited from healthcare facilities across four islands of Cabo Verde: Santiago, Fogo, São Vicente, and Boavistaas part of a cross-sectional study combining interview -based data collection with retrospective extraction of clinical data. Participants were included if they were recently diagnosed with HIV-1, had not received antiretroviral therapy, and had available CD4+ T lymphocyte count data. Individuals with incomplete clinical records were excluded from the analysis.

### 2.2 Data collection

For data collection, a structured questionnaire was administered through face-to-face interviews with patients who consented to participate in the study. The questionnaire covered sociodemographic, behavioural, and clinical information.

Clinical data were obtained from the participants' medical records, including CD4+ T lymphocyte count, viral load, and the presence of opportunistic infections at diagnosis to determine the stage of HIV infection. In parallel, HIV-1 genetic sequences, derived from a study on transmitted antiretroviral drug resistance (41), were analysed to calculate the nucleotide ambiguity rate, providing a complementary assessment to the clinical classification of infection duration.

### 2.3 Definition and criteria for late HIV diagnosis

According to the updated consensus definition proposed by Croxford et al. (23), late HIV diagnosis is defined as a diagnosis of HIV with a CD4+ T-lymphocyte count <350 cells/μL at the time of diagnosis or the presence of an AIDS-defining condition, regardless of the CD4+ T-cell count.

### 2.4 Genetic ambiguities

Sequences of the HIV-1 protease and reverse transcriptase genes, obtained by standard genotyping, were used. Nucleotide ambiguity rates were calculated using an RStudio script (v. 2023.03.1 + 446), which determines the sequence length, identifies ambiguous nucleotides, and computes the ambiguity of each sequence as the difference between the original length and the length after removal of ambiguities, divided by the original length. Infection duration was estimated based on the nucleotide ambiguity rate, with recent infection defined as ≤0.47% and chronic infection as >0.47% (12, 35), based on the progressive accumulation of mutations during viral replication over time. However, this interpretation should be approached cautiously, as viral diversity may also be influenced by transmission dynamics.analysis.

### 2.5 Statistical analysis

The statistical analysis was performed using IBM SPSS Statistics, version 27 (IBM Corp., Armonk, NY, USA). Descriptive statistics were presented as absolute (*N*) and relative (%) frequencies. Chi-square and Fisher's exact tests were used to examine associations between variables. Factors associated with HIV-1 late diagnosis were assessed using univariable and multivariable logistic regression. Variables considered for inclusion in the multivariable model were selected based on epidemiological relevance, statistical association in univariable analyses, completeness of data, and absence of conceptual overlap with the outcome definition. Unadjusted and adjusted odds ratios (aOR) with 95% confidence intervals (CI) were reported. *P*-values <0.05 were considered statistically significant.

### 2.6 Ethics approval

The study was conducted following the Declaration of Helsinki and approved by the National Health Research Ethics Committee of Cabo Verde (Reference no. 13/2018) and the National Data Protection Commission (Reference no. 192/2018). Written informed consent was obtained from all study participants. The investigators guaranteed the anonymity and confidentiality of participants at all stages of the study.

## 3 Results

### 3.1 Characteristics of study population

The sociodemographic, clinical and genetic characteristics of the study population included in the analyses of late HIV diagnosis and non-late HIV diagnosis are summarized in Table 1. A total of 135 newly diagnosed, treatment-naïve individuals were included. Among them, 56.3% (*n* = 76) were classified as late HIV diagnosis and 43.7% (*n* = 59) as non-late HIV diagnosis. The population comprised 51.9% (*n* = 70) females and 48.1% (*n* = 65) males. Among individuals with late HIV diagnosis, 53.9% (*n* = 41) were male and 46.1% (*n* = 35) were female. Among individuals reporting homosexual contact (*n* = 5) identified in the study, 3.9% (*n* = 3) were classified as having a late HIV diagnosis.

**Table 1:** Sociodemographic and clinical characteristics of the participants included, and prevalence of late HIV diagnosis and non-late HIV diagnosis.

Characteristics of the participants	Overall, *n* (%)	Late HIV diagnosis *n* (%)	Non-late HIV diagnosis *n* (%)	*p*-value
Total, *n* (%)	135 (100.0)	76 (56.3%)	59 (43.7%)	–
Sex, *n* (%)				0.126
Male	65 (48.1)	41 (53.9%)	24 (40.7%)	
Female	70 (51.9%)	35 (46.1%)	35 (59.3%)	
Age groups at diagnosis, years, *n* (%)				
Median [1st, 3rd quartile] (IQR)	47 [35–57]	49.5 [39–59]	43 [33–54]	**0** **.** **040**
≤30	34 (25.2%)	17 (22.4%)	17 (28.8%)	0.224
31–40	29 (21.5%)	12 (15.8%)	17 (28.8%)	
41–50	34 (25.2%)	22 (28.9%)	12 (20.3%)	
51–60	25 (18.5%)	17 (22.4%)	8 (13.6%)	
>60	13 (9.6%)	8 (10.5%)	5 (8.5%)	
Marital status at the time of diagnosis, *n* (%)				**0** **.** **008**
Married	33 (24.4%)	12 (15.8%)	21 (35.6%)	
Single	102 (75.6%)	64 (84.2%)	38 (64.4%)	
HIV exposure category, *n* (%)				0.240
Heterosexual contact	126 (93.3%)	69 (90.8%)	57 (96.6%)	
Homosexual contact	5 (3.7%)	3 (3.9%)	2 (3.4%)	
Other	4 (3.0%)	4 (5.3%)	0 (0.0%)	
Education, *n* (%)				0.375
Illiterate/only literate	27 (20.0%)	15 (19.7%)	12 (20.3%)	
Elementary school	67 (49.6%)	42 (55.3%)	25 (42.4%)	
Secondary education	39 (28.9%)	18 (23.7)	21 (35.6%)	
University	2 (1.5%)	1 (1.3%)	1 (1.7%)	
Residence, *n* (%)				0.660
Santiago	110 (81.5%)	63 (82.9%)	47 (79.7%)	
Other island	25 (18.5%)	13 (17.1%)	12 (20.3%)	
Occupation, *n* (%)				0.128
Employee	116 (85.9%)	64 (84.2%)	52 (88.1%)	
Unemployed	17 (12.6%)	12 (15.8%)	5 (8.5%)	
Student	2 (1.5%)	0 (0.0%)	2 (3.4%)	
CD4+ T lymphocyte count, cells/μL				
Median [1st; 3rd quartile]	309 [143, 529]	160 [71.5–250.5]	548 [442.5–759.0]	**<0** **.** **001**
Viral load at diagnosis, copies/mL				
<4.0	51 (37.8%)	20 (26.3%)	31 (52.5%)	
4.1–5.0	25 (18.5)	13 (17.1%)	12 (20.3%)	**0** **.** **003**
≥5.0	36 (26.7%)	28 (36.8%)	8 (13.6%)	
Unknown	23 (17.0%)	15 (19.7%)	8 (13.6%)	
Opportunistic infection				
Yes	60 (44.4%)	47 (78.3%)	13 (21.7%)	**<0.001**
No	75 (55.6%)	29 (38.7%)	46 (61.3%)	
Genetic ambiguities				
Overall	60 (100%)	33 (55.0%)	27 (45.0%)	0.387
Recent infection (≤0.47%)	41 (68.3%)	**21 (63.6%)**	20 (74.1%)	
Chronic infection (≥0.47%)	19 (31.7%)	12 (36.4%)	**7 (25.9%)**	

*P*-values were obtained using the chi-square test and the Fisher's exact test.

Bold values indicate statistically significant results (*p* < 0.05).

The median age at diagnosis was 47 years [interquartile range (IQR) 35–57]. Individuals with late HIV diagnosis were significantly older than those without late HIV diagnosis (median 49.5 vs. 43 years, *p* = 0.040). The frequency of late HIV diagnosis was higher among individuals aged 41–50 years (28.9%, *n* = 22) and 51–60 years (22.4%, *n* = 17).

The majority of participants were single (75.6%, *n* = 102), with this condition being significantly more prevalent among those with HIV-1 late HIV diagnosis, of whom 84.2% (*n* = 64) were single, compared to only 15.8% (*n* = 12) who were married (*p* = 0.008).

Analysis of viral load at diagnosis revealed statistically significant differences between the groups. A higher proportion of those with HIV-1 late HIV diagnosis had a viral load ≥5.0 log copies/mL (36.8%, *n* = 28) compared to non-late HIV-1 diagnosis, of whom only (13.6%; *n* = 8) had viral load ≥5.0 log copies/mL (*p* = 0.003).

Opportunistic infections were more frequently observed among individuals with HIV-1 late HIV diagnosis. Overall, 44.4% of participants (*n* = 60) presented at least one opportunistic infection. Among these, 78.3% (*n* = 47) were in the late HIVdiagnosis group and 21.7% (*n* = 13) in the non-late HIV diagnosis group. The most frequent opportunistic infections were candidiasis (26; 43.3%), herpes zoster (12; 20.0%), chronic diarrhea (10; 16.7%), and pulmonary tuberculosis (9; 15.0%) (Supplementary Table S1).

A total of 60 HIV-1 sequences were successfully analysed for genetic ambiguities. Among these, 68.3% (*n* = 41/60) were classified as recent infection (≤0.47% ambiguity) and 31.7% (*n* = 19/60) as chronic infection (≥0.47% ambiguity). Comparison with CD4+ T lymphocyte counts showed concordance in 51.7% (*n* = 31) of cases, while 48.3% (*n* = 29) displayed discordant results. No statistically significant differences were observed in other variables analysed.

The associations between clinical and demographic variables and late HIV diagnosis are presented in Table 2. In the univariable analysis, marital status at diagnosis and viral load were significantly associated with late HIVdiagnosis. In the multivariable logistic regression analysis, these associations remained significant. Single individuals had a higher probability of having late HIV diagnosis compared to married individuals (aOR: 2.791; 95% CI: 1.125–6.922; *p* = 0.027). Similarly, participants with a viral load ≥5.1 log copies/mL were more likely to have late HIV diagnosis than those with a viral load <4.0 log copies/mL (aOR: 5.242; 95% CI: 1.802–15.251; *p* = 0.002).

**Table 2:** Logistic regression analysis of factors associated with late HIV diagnosis among newly diagnosed, treatment-naïve individuals in Cabo Verde (2018–2019).

Variables	Unadjusted		Final model	
	OR (95% CI)	*p*-value	aOR (95% CI)	*p*-value
Sex				
Male	0.585 (0.294–1.165)	0.127	0.697 (0.299–1.622)	0.402
Female	Ref		Ref	
Age groups at diagnosis, years				
≤30	Ref		Ref	
31–40	1.417 (0.522–3.847)	0.494	1.575 (0.513–4.829)	0.427
41–50	0.545 (0.206–1.443)	0.222	0.774 (0.216–2.778)	0.695
51–60	0.471 (0.160–1.380)	0.170	0.488 (0.125–1.910)	0.303
>60	0.625 (0.170–2.302)	0.625	0.527 (0.103–2.683)	0.440
Marital status at the time of diagnosis				
Married	Ref		Ref	**0** **.** **027**
Single	2.947 (1.305–6.658)	**0** **.** **009**	2.791 (1.125–6.922)	
Viral load, copies/mL at diagnosis				
<4.0	Ref		Ref	
4.1–5.0	1.679 (0.640–4.408)	0.293	1.677 (0.605–4.652)	0.320
≥5.0	5.425 (2.065–14.255)	**<0** **.** **001**	5.242 (1.802–15.251)	**0** **.** **002**

Univariable and multivariable logistic regression analyses were performed. Adjusted odds ratios (aOR) with 95% confidence intervals (CI) are shown.

Bold values indicate statistically significant results (*p* < 0.05).

## 4 Discussion

Despite significant progress in HIV prevention and treatment, late HIV diagnosis continues to represent a serious public health problem globally. Late HIV diagnosis is associated with higher mortality (42, 43), reduced life expectancy (44) and higher medical costs (20). Understanding the prevalence of late HIV diagnosis is essential, as it constitutes a crucial indicator for evaluating the effectiveness of prevention programmes and testing strategies (45).

In the present study, the prevalence of late HIV diagnosis among HIV-1 cases newly diagnosed in 2018–2019 across the four islands included (Santiago, Fogo, Boavista, and São Vicente) was 56.3% (76/135), with 57.3% (63/110) in Santiago, a value identical to that reported by the only previous study conducted in the country, which included Santiago only (16). The remaining three islands had a combined prevalence of late HIV diagnosis of 52%, indicating that, although Santiago accounts for the majority of HIV-1 cases in Cabo Verde (70.6%) (37), late HIV diagnosis occurs consistently across different regions.

Our results are consistent with the estimates for Africa, where late HIV diagnosis ranges from 35% to 65% (46–48), and are in line with those reported in European studies, which range from 48.1% to 55.2% (12, 49, 50). The high proportion (56.1%) of late HIV diagnosis observed in this study represents a significant challenge to achieving the UNAIDS 95-95-95 targets, which are essential for ending the HIV/AIDS epidemic by 2030. Late HIV diagnosis may be associated with multiple factors, including social stigma (51), diagnosis at advanced stages (8) and loss to follow-up within the healthcare system (52, 53). In Cabo Verde, 29% of men and 41% of women hold discriminatory attitudes towards PLHIV (40). In the specific context of HIV, stigma often keeps individuals from seeking an early diagnosis and adherence to treatment. The lack of awareness of one's serological status also contributes to delays in the initiation of ART (54). Despite the free availability of HIV diagnostic tests in the country, the proportion of HIV-1 late HIV diagnosis remains high (56%), emphasizing the importance of promoting early diagnosis and reducing stigma.

The analysis of factors associated with late HIV diagnosis revealed that the variables such as marital status and viral load at diagnosis were significantly associated with late HIV diagnosis. Single individuals exhibited a higher risk of late HIV diagnosis compared to those who were married. This association may reflect differences in social support and the influence of stigma, which continues to be a significant barrier to early diagnosis and access to treatment. HIV-related stigma remains a crucial factor in the persistence of the pandemic, negatively affecting therapeutic outcomes and the quality of life of PLHIV (55–58). The influence of stigma is also correlated with adverse clinical outcomes, such as mental health disorders and failure to achieve viral suppression (59). PLHIV who experience stigma are more than twice as likely to present late for ART initiation and other HIV-related services (51). These findings underscore the importance of implementing effective stigma-reduction strategies and promoting equitable access to healthcare.

Regarding viral load, this study revealed a significant association between high viral load and late HIV diagnosis. These findings are consistent with previous evidence indicating that individuals with advanced untreated HIV disease have higher viral loads and rapid disease progression, which increases the risk of transmission and mortality (60–62). The rise in HIV-RNA levels is directly related to a more rapid decline in CD4+ T lymphocyte count, which leads to a continued decline of these cells and the progression of the infection in the absence of ART. Therefore, this will allow for continuous viral replication and, consequently, the deterioration of the immune system (63–65). This high viral load not only increases the risk of therapeutic failure but also drives intense viral replication, which can lead to the divergence of the viral population, forming complex quasispecies. Resistant mutants may then be selected once ART is initiated, making infection control even more challenging.

This finding reinforces the critical importance of early diagnosis and the immediate initiation of ART, since treatment delays can result in greater immunological damage and a poorer clinical prognosis, in line with WHO guidelines.

Finally, this study observed a higher frequency of opportunistic infections of opportunistic infections, which is consistent with the expected immunosuppression associated with low CD4+ T lymphocyte count at diagnosis. These infections significantly contribute to morbidity and mortality among PLHIV (66, 67). To mitigate these impacts, the WHO recommends a package of interventions, including screening, treatment and/or prophylaxis for opportunistic infections, rapid initiation of ART, and strategies to enhance treatment adherence (2). Implementing these measures is essential for effective clinical management and for reducing the disease burden.

The analysis of genetic ambiguity in 60 participants allowed the assessment of infection recency, exploring whether viral diversity could provide complementary information to CD4+ T-cell counts. Our findings are consistent with previous studies (34, 35, 68), suggesting that lower proportion of ambiguous nucleotides in HIV-1 sequences may be associated with more recent HIV-1 infection. Our results suggest that genetic ambiguity thresholds can be applied to non-B subtypes, predominant in Cabo Verde, to support the estimation infection recency and epidemiological monitoring, in line with evidence from other studied populations (34, 68, 69).

The combined analysis of CD4+ T-cell counts and ambiguity rates revealed that 48.3% of individuals had discordant results, indicating that not all follow the expected correlation between immunological progression and viral diversity. Cases with high CD4+ counts and high ambiguity may reflect multiple infections, slow disease progression, or effective immune responses, whereas low CD4+ counts with low ambiguity may indicate rapid progression, recent infection, or monoclonal infection (31, 33–35, 68). These findings emphasize that CD4+ counts alone and ambiguity rates alone are both insufficient to determine the stage of infection. The observed heterogeneity in HIV-1 disease progression underlines the need for public health strategies that integrate multiple biomarkers, enabling earlier detection and more effective monitoring of HIV-1 infection.

Although variables such as sex, age, residence, education level, and occupation were not significantly associated with late HIV diagnosis in our study, analysing these factors remains relevant for understanding determinants of delayed HIV diagnosis and treatment initiation. Differences in study period, population characteristics, and methodology may explain these discrepancies with previous findings.

A prior study in Cabo Verde (16) reported an association between older age, education level, the time between HIV diagnosis and the start of ART and late HIV diagnosis, suggesting a possible impact of these characteristics on disease progression and access to health care.

Although no significant association was identified between age groups at diagnosis and late HIV diagnosis (*p* = 0.224), a higher proportion of late HIV diagnosis was observed in the 41–50 age group (16.3%) and the 51–60 age group (12.6%), compared to those under 30 years old (12.6%). Similar results were reported by Moreira et al. (2016) in Cabo Verde, as well as by other international studies that consistently indicated that older age is a factor associated with late HIV diagnosis (12, 53, 70–72). The mistaken perception that older individuals are at lower risk of HIV infection may contribute to the reduced demand for testing, which results in late HIV diagnosis. Of note, many of these individuals may have acquired the infection at a younger age, only being diagnosed in more advanced stages of the disease. Therefore, it is essential to promote HIV screening in all age groups, even in the absence of symptoms, with the aim of reducing the occurrence of late HIV diagnosis and improving outcomes on the continuum of care.

Men were more frequently classified as late HIV diagnosis (53.9%) than women (46.1%), suggesting that women had more opportunities to be tested for HIV. This fact may be related to the policy of mandatory screening during prenatal consultations as part of the prevention of vertical transmission of HIV. This measure facilitates early detection of infection in women, reducing the proportion of late HIV diagnosis (73). Additionally, various studies indicate lower acceptance of HIV testing by men (74, 75), leading to a greater tendency towards late-stage diagnosis. These data highlight the need to implement specific strategies for targeting early HIV infection diagnosis in men in Cabo Verde, aiming to increase screening and early diagnosis.

HIV-1 Late diagnosis represents a significant barrier to achieving the UNAIDS 95-95-95 targets. Therefore, the intensification of early testing programmes, especially those targeting populations with similar characteristics as described as individuals with late HIV diagnosis, is highly important to improve timely diagnosis and effective clinical follow-up for HIV.

### 4.1 Limitations

This study provides valuable insights into HIV-1 late diagnosis in Cabo Verde by integrating clinical, demographic, and molecular data. Some limitations should be noted, including the relatively small sample size, the inclusion of only newly diagnosed patients, incomplete clinical data for some individuals, and the limited number of sequences available for genetic ambiguity analysis, which may have limited the statistical power of some analyses. Nevertheless, the findings make a meaningful contribution to understanding HIV epidemiology in the country and support the development of evidence-based strategies to reduce late HIV diagnosis.

## 5 Conclusion

HIV-1 late diagnosis remains highly prevalent in Cabo Verde, affecting more than half of newly diagnosed individuals. In this study, being single and having a high viral load at diagnosis were independently associated with late HIV diagnosis. Late HIV-1 diagnosis remains the main risk factor for mortality and contributes to ongoing HIV transmission. Early diagnosis and treatment are essential to maximise the individual benefits of ART and to improve public health. The integration of clinical, demographic, and genetic data, including viral ambiguity analysis, provides a more comprehensive understanding of infection duration and disease progression. These findings highlight the urgent need for targeted strategies to promote early HIV testing, ensure timely linkage to care, and reduce late diagnoses, thereby improving both individual outcomes and public health, in line with the 95-95-95 targets for HIV/AIDS elimination by 2030. Further studies are needed to better understand the drivers of late HIV diagnosis, including the impact of stigma and discrimination on the decision to undergo HIV testing in Cabo Verde.

## Data Availability

The original contributions presented in the study are included in the article/Supplementary Material, further inquiries can be directed to the corresponding author.
