# Supplementary material for: HIV-1 late diagnosis in Cabo Verde: associated factors and implications for preventive strategies
Source: Front Epidemiol. 2026 Jun 11;6:1861884. doi: 10.3389/fepid.2026.1861884 (PMC13294154; doi:10.3389/fepid.2026.1861884)
Supplement: Supplementary file 1 [file Table1.docx]

Supplementary table S1: Distribution of opportunistic infections among individuals with late HIV-1 diagnosis.

This table presents the distribution of opportunistic infections among participants classified as late HIV-1 diagnosis. Percentages are calculated based on the total number of individuals with at least one opportunistic infection (n = 60).

| **Opportunistic Infection** | **n (%)** |
| --- | --- |
| Candidiasis | 26 (43.33%) |
| Herpes Zoster | 12 (20.0%) |
| Chronic Diarrhoea | 10 (16.67%) |
| Pulmonary Tuberculosis | 9 (15.0%) |
| Pneumonia (non-specific) | 5 (8.33%) |
| Pneumocystis pneumonia | 6 (10.0%) |
| Herpes Simplex | 3 (5.0%) |
| Kaposi sarcoma | 2 (3.33%) |
| Cerebral Toxoplasmosis | 2 (3.33%) |
